# Restarting cataract surgery during the COVID-19 pandemic; a prospective study analysing 30 day outcomes after elective cataract surgery in the United Kingdom

**DOI:** 10.1186/s12886-021-01936-0

**Published:** 2021-04-09

**Authors:** Francis Carr, Paras Agarwal, Harmehak Narula, Tiran S. Keragala, Samer Elshikh Hassan Awwad, Ahmed Roble, Vinod Gangwani

**Affiliations:** Ophthalmology Department, Ashford & St Peter’s Hospitals NHS Foundation Trust, Ashford, UK

**Keywords:** Cataract, Cataract surgery, Phacoemulsification, COVID-19, Coronavirus

## Abstract

**Background:**

Cataract is a significant cause of preventable blindness in the United Kingdom and worldwide. Prior to the COVID-19 pandemic, cataract surgery was the most commonly performed operation by the National Health Service in the United Kingdom. The aim of this study is to evaluate the safety of elective cataract surgery performed in the United Kingdom in a COVID-19 free hospital during the COVID-19 pandemic.

**Methods:**

Single centre prospective observational cohort study of consecutive patients undergoing elective cataract surgery in the United Kingdom over a 3 month period from May to August 2020. Electronic medical records were reviewed and patients were contacted at 30 days post-operatively. Data collection included symptoms suggestive of COVID-19 infection, hospital admission, mortality, intra-operative and post-operative surgical complications.

**Results:**

A total of 649 elective cataract surgeries were performed. Two patients (0.3%) developed worsening dyspnoea during the 30 day post-operative period, but tested negative for COVID-19 infection. Three patients (0.5%) were hospitalised, unrelated to COVID-19 infection, of which one patient (0.2%) passed. Four patients (0.6%) suffered posterior capsular rupture. 601 (93.2%) had no post-operative complications.

**Conclusion:**

This study demonstrates a safe model for the resumption of elective cataract surgery during the COVID-19 pandemic, providing strict infection control measures are in place.

## Introduction

Prior to the coronavirus disease 2019 (COVID-19) pandemic, cataract surgery was the most commonly performed operation in the United Kingdom’s National Health Service (NHS) [1]. The World Health Organisation (WHO) estimates cataract is the second largest cause of treatable visual impairment globally [2]. In the United Kingdom, an estimated 380,000 people are living with sight loss due to cataract [3].

COVID-19 was declared a Public Health Emergency of International Concern by WHO on 30 January 2020 [4]. Since its outbreak, it has brought significant harm and challenges to over 200 countries and regions around the world. To date, there have been over 50 million confirmed cases of COVID-19 and over one million deaths reported to WHO [5].

In response to rising COVID-19 cases in the United Kingdom, NHS England and NHS Improvement announced COVID-19 a Level 4 National Incident on 17 March 2020 [6]. NHS trusts were instructed to suspend elective services to minimise virus transmission and augment capacity to cope with a surge in COVID-19 infections and its demand on hospital resources. On 28 March 2020, the Royal College of Ophthalmologists (RCOphth) recommended eye units postpone all elective eye operations and non-urgent outpatient clinics [7]. Understandably, cataract surgery faced significant disruption. On 16 April 2020, the RCOphth issued guidance regarding the prospective resumption of cataract surgery in order to ensure a safe environment for patients and staff, acknowledging the necessity to address the resultant large backlog in cases and provide patients with a timely service [8]. Failure to do so would compromise the quality of life of many patients [9].

Currently, there is no available data investigating safety following the resumption of cataract surgery during the COVID-19 pandemic. This prospective study provides data regarding patients undergoing elective cataract surgery performed in a COVID-19 free hospital in the United Kingdom.

## Methods

This single centre, prospective, observational cohort study analysed patients who had elective cataract surgery, under local and general anaesthesia, at Ashford Hospital (part of the Ashford and St Peter’s NHS Foundation Trust) in the United Kingdom. Ashford Hospital has been a COVID-19 free hospital throughout the COVID-19 pandemic, as defined by no inpatients with suspected or proven COVID-19 infection. Consecutive patients undergoing cataract surgery over a 3 month period during the COVID-19 pandemic, from 19 May 2020 (when cataract services resumed at the aforementioned hospital) to 18 August 2020, were included. Data collection was routine, with no resultant alteration in clinical practice. The Institutional Review Board at Ashford and St Peter’s NHS Foundation Trust reviewed and approved the study protocol (TASCCOPHTH (2020-05)). All study conduct adhered to the Declaration of Helsinki for the use of human participants in biomedical research.

After full discussion of the potential benefits and risks of undergoing cataract surgery during the COVID-19 pandemic, over the phone, patients were offered cataract surgery on the basis of waiting list time and clinical urgency. Patients were not excluded from selection due to age or comorbidities. All patients signed a consent form acknowledging the potential risk of contracting COVID-19 infection during their visit to the hospital.

All patients were screened with a COVID-19 nasopharyngeal swab test (testing for severe acute respiratory syndrome coronavirus 2 RNA) 72 h before the day of surgery, and were allowed to proceed providing it was negative. The testing was performed at a drive-through test site, with the patient required to remain in their car during the testing process. Following national guidance, patients were initially instructed to self-isolate for 14 days before the operation for surgery. A change in National Institute for Health and Care Excellence (NICE) recommendations [10] resulted in a reduction in the self-isolation period to 3 days following their pre-operative COVID-19 swab, which was implemented 5 August 2020 onwards. Patients were not instructed to isolate following their operation.

Patients had staggered admission times to strictly maintain a two metre distance between patients, with five patients listed for each morning and afternoon list. No accompanying persons were allowed into the hospital with the patient, unless required due to cognitive impairment. On entering the hospital, temperature was monitored using a non-contact handheld cutaneous infrared thermometer and the patients were screened for symptoms suggestive of COVID-19 infection. Patients with symptoms or a temperature > 37.8 degrees Celsius precluded entry into the hospital. Patients were instructed to don a new sterile surgical mask whilst they were in hospital, which was removed temporarily during the operation, when a sterile drape was placed over the head. The mask was replaced immediately following completion of the operation and placement of the protective eye shield.

Local policy was implemented to minimise potential risk of spreading infection. Each team member wore appropriate personal protective equipment, including surgical masks, aprons and gloves whilst operating and coming into contact with patients. No changes were made to personnel performing cataract surgery, with trainee lists continuing as normal, although the majority of operations were performed by consultant ophthalmologists. Operating surgeons did not necessarily spend all day in theatre, with some attending clinic in the morning, followed by theatre in the afternoon, and vice-versa.

Ophthalmology theatres were not separate from theatres used by other specialities. No changes were made to theatre airflow conditions (laminar flow). For patients requiring general anaesthesia, intubation and extubation occurred in theatre, with a minimum of 7 min before other staff could enter, to reduce the risk of aerosol transmission.

Routine post-operative care comprised of two phone calls from trained ophthalmology nursing staff at post-operative days one and 14, followed by an optician review at 6 weeks. Routine face-to-face appointments could be arranged at the discretion of the operating surgeon. Patients were issued with a 24 h telephone number to contact the ophthalmology department (within office hours) or on-call ophthalmologist (outside of office hours) if post-operative complications arose and they required a face-to-face review. Similarly, if nursing staff phone calls identified a post-operative issue requiring a face-to-face review, then that appointment could be arranged at their discretion.

Data for the study was collected 30 days following cataract surgery. Electronic health records were reviewed and patients were contacted via telephone. If patients were unable to communicate via telephone then data was collected via their main carer.

The primary outcome of the study was swab proven COVID-19 infection within the 30 day post-operative period. Secondary outcomes included COVID-19 symptoms (high temperature, cough, dyspnoea, coryzal symptoms, anosmia and systemic upset), hospitalisation, ICU admission and mortality.

Intra-operative complications were recorded, by reviewing the operation report. Post-operative follow-up was analysed, including arranged follow-up (whether via telephone or face-to-face), complications arising within the 30 day period, the number of reviews within the 30 day period and the number of subsequent operations.

Patient experience data was collected. This encompassed a questionnaire over the telephone, using a five point Likert scale. Each question was analysed independently.

Statistical analysis was performed using SPSS Statistics Version 26 (IBM, USA). Quantitative endpoints are presented as mean ± standard deviation. Qualitative endpoints are presented as numbers and the percentage of each modality.

## Results

The patient demographics are demonstrated in Table 1. All patients were able to be contacted via telephone, alongside a review of their electronic medical records.

**Table 1:** Demographic data

Eye	
Right	322 (49.6%)
Left	327 (50.4%)
First	353 (54.3%)
Second	296 (45.6%)
Anaesthesia	
Local	646 (99.5%)
General	3 (0.5%)
Age (years)	
Mean (SD)	73.7 (9.61), min 21 max 97
Age group	
≤ 45	2 (0.3%)
46–64	120 (18.5%)
65–74	198 (30.5%)
75–84	261 (40.2)
≥ 85	68 (10.5%)
Sex	
Male	272 (42.0%)
Female	377 (58.0%)
Ethnicity	
Non-BAME	469 (72.3%)
BAME	180 (27.7%)
Body Mass Index (kg/m^2^)	
Mean (SD)	25.7 (4.60), min 14.7 max 58.3
< 18.5	14 (2.2%)
18.5–24.9	317 (48.8%)
25.0–34.9	297 (45.8%)
35.0–44.9	18 (2.8%)
≥ 45	3 (0.5%)
Comorbidities	
Active malignancy	31 (4.8%)
Cardiac disease (including arrhythmias, ischaemic heart disease and cardiac failure)	158 (24.3%)
Cerebrovascular disease	30 (4.6%)
Chronic kidney disease	32 (4.9%)
Chronic lung disease (including asthma and chronic obstructive pulmonary disease)	101 (15.6%)
Diabetes mellitus	173 (26.7%)
Hypertension	344 (53.0%)
Taking immunosuppressive medication (including prednisolone)	18 (2.8%)

Over the 3 month period, from 19 May 2020 to 18 August 2020, 649 elective cataract surgeries were performed on 631 patients, with 18 patients having both eyes operated on within the study timeframe. 322 (49.6%) of the operations were on the right eye, 327 (50.4%) were on the left eye, 353 (54.3%) were the first eye, 296 (45.6%) were the second eye. The mean age (± SD) was 73.7 years ±9.61, 272 (42.0%) were male eyes, 377 (58.0%) were female eyes. 180 (27.7%) patients identified as Black, Asian and minority ethnic (BAME). With regards to comorbidities, hypertension was the most common, 344 (53.0%), followed by diabetes mellitus (173, 26.7%) and cardiac disease (158, 24.3%).

Regarding anaesthesia, nearly all operations (646, 99.5%) were performed under local anaesthesia. 638 (98.3%) had no intraoperative complications (Table 2). The most common intraoperative complication was posterior capsular rupture (4, 0.6%), followed by zonular dialysis (3, 0.5%). Most follow-up was performed over the phone, with 44 operations (6.8%) requiring a face-to-face review (Table 3), either due to an intra-operative complication, or an associated procedure performed during the operation, such as a dexamethasone implant, iStent implant or toric lens insertion.

**Table 2:** Intraoperative complications

Intraoperative complications	
None	638 (98.3%)
Posterior capsular rupture	4 (0.6%)
Zonular dialysis but not vitreous prolapse	3 (0.5%)
Anterior capsular tear out	2 (0.3%)
Descemet’s tear	1 (0.2%)
Dropped nucleus	1 (0.2%)

**Table 3:** Follow-up and post-operative complications

Planned post-operative follow-up	
Phone call alone	605 (93.2%)
Face-to-face appointment (e.g. toric lens, dexamethasone implant, iStent, intra-op complication)	44 (6.8%)
Post-operative complications within 30 day period	
None	601 (92.6%)
Floaters/uncomplicated posterior vitreous detachment	11 (1.7%)
Corneal oedema	8 (1.2%)
Ocular surface disorder	8 (1.2%)
Blurred vision but no abnormality detected	5 (0.8%)
Raised intraocular pressure	5 (0.8%)
Macular oedema	4 (0.6%)
Dysphotopsia	2 (0.3%)
Central retinal vein occlusion	2 (0.3%)
Posterior capsular opacification	1 (0.2%)
Refractive surprise	1 (0.2%)
Retained lens matter	1 (0.2%)
Face-to-face appointment within 30 days post-op (including routine appointments)	
No	565 (87.1%)
Yes	84 (12.9%)
	Planned: 44 (6.8%)
	Unplanned: 40 (6.2%)
	Number of appointments
	1: 60 (9.2%)
	2: 9 (1.4%)
	3: 10 (1.5%)
	4: 4 (0.6%)
	5: 1 (0.2%)
Further operation within 30 days post-op	3 (0.5%) Vitrectomy + secondary IOL insertion: 2 (0.3%) AC washout: 1 (0.2%)
Duration between symptoms and being reviewed in clinic	
Average (SD)	4.5 (3.47)
Range	0-14d
0 days	5 (12.5%)
1–3 days	14 (35%)
4–7 days	16 (40%)
8–14 days	5 (12.5%)
Unplanned face-to-face appointments *‘I had difficulty being seen regarding my post-operative symptoms’*	40 (6.2%)
Strongly agree	4 (10%)
Agree	2 (5%)
Neutral	8 (20%)
Disagree	6 (15%)
Strongly disagree	20 (50%)

Forty patients (6.2%) required an unscheduled face-to-face review in the 30 day post-operative period. Of those, 5 (12.5%) reviews were on the same day as the patient reporting their symptoms, 14 (35%) were within 1–3 days, 16 (40%) were within 4–7 days and 5 (12.5%) were within 8–14 days. 20 (50%) strongly disagreed with the statement ‘it was difficult to arrange an unscheduled face-to-face appointment’, 4 (10%) strongly agreed.

84 (12.9%) patients required a face-to-face review in clinic within 30 days of their operation, including both scheduled and unscheduled. 60 (9.2%) required one review in total, one patient (0.2%) required five reviews.

48 (7.4%) post-operative complications were identified within 30 days, the most common being newly recognised floaters (11, 1.7%), followed by corneal oedema and ocular surface disorders, both in 8 eyes (1.2%). Three patients (0.5%) required further surgery within the 30 day period, two (0.3%) of which were vitrectomy and secondary intraocular lens (IOL) insertion, the other (0.2%) being an anterior chamber washout due to retained lens matter.

Ninety-six patients (14.8%) continued shielding following the operation (Table 4). Two patients (0.3%) reported having suffered COVID-19 symptoms within the 30 day post-operative period, both of which were dyspnoea and both of which tested negative for COVID-19. Of the entire cohort, three patients (0.5%) had COVID-19 nasopharyngeal swabs performed, of which all were negative. Those three patients had their swabs performed during admission to hospital. One patient was admitted for investigation of vertigo, another for management of an exacerbation of combined cardiac and renal failure and the last patient due to an exacerbation of cardiac failure secondary to aortic valve stenosis (who sadly passed within the 30 day post-operative period, his next-of-kin supplied data). The medical teams deemed all three presentations unrelated to COVID-19 infection.

**Table 4:** Symptomatic COVID-19 infection rates post-operatively

30 day post-operative period	
Shielding	Yes: 96 (14.8%)
	No: 553 (85.2%)
COVID-19 symptoms	Yes: 2 (0.3%), both worsening dyspnoea
	No: 647 (99.7%)
COVID-19 swab performed	Yes: 3 (0.5%), all negative
	No: 646 (99.5%)
Hospital admission	Yes: 3 (0.5%)
	No: 646 (99.5%)
ICU admission	No: 649 (100%)
Mortality	Yes: 1 (0.2%)
	No: 648 (99.8%)

Patient experience data was collected from all patients (Table 5), barring the one mortality (648 in total). 507 (78.2%) strongly agreed with the statement that they were ‘happy with their decision to proceed with cataract surgery’, 2 patients (0.3%) strongly disagreed, both of which had suffered complications related to their surgery. The majority of patients either disagreed (123, 19.0%) or strongly disagreed (366, 56.5%) with the statement that they were concerned they may have caught COVID-19 due to attending hospital for the cataract surgery. The vast majority strongly agreed (578, 89.2%) or agreed (68 (10.5%) with the statement that there were adequate infection control measures in hospital to prevent transmission of COVID-19.

**Table 5:** Patient experience

*‘I was happy with my decision to proceed with cataract surgery during the COVID-19 pandemic’*	
Strongly agree	507 (78.2%)
Agree	109 (16.8%)
Neutral	24 (3.7%)
Disagree	6 (0.9%)
Strongly disagree	2 (0.3%)
*‘I was concerned I may have acquired COVID-19 infection due to attending hospital for my cataract surgery’*	
Strongly agree	38 (5.9)
Agree	50 (7.7%)
Neutral	71 (11.0%)
Disagree	123 (19.0%)
Strongly disagree	366 (56.5%)
*‘I believe there were adequate infection control measures in hospital to prevent transmission of COVID-19’*	
Strongly agree	578 (89.2%)
Agree	68 (10.5%)
Neutral	1 (0.2%)
Disagree	0 (0.0%
Strongly disagree	1 (0.2%)

## Discussion

As far as we are aware, this is the first study demonstrating prospectively collected data on the safety of elective cataract surgery performed during the current COVID-19 pandemic. Due to the multitude of cancelled elective operations, the backlog has been rapidly accumulating. Numerous recommendations have been provided in order to safely resume elective services [8, 10–12]. We provide evidence of a safe model for the resumption of elective cataract surgery in a COVID-19 era.

None of our cohort had swab proven COVID-19 infection in the 30 day post-operative period. Regarding symptoms, very few patients reported COVID-19 related symptoms following the operation. Patient recollection is fallible, although we believe that patients would be particularly mindful of them in current times. If significant enough to warrant presentation to hospital, then we were able to monitor for this independently by reviewing electronic medical records, although this would not detect patients who had travelled out-of-area and subsequently became unwell. Patients may be reticent to disclose symptoms due to the concern that they may subsequently be asked to self-isolate or it may in some way affect their post-operative care (although the contrary was stated during data collection). Our study was the unable to detect asymptomatic infection rates in patients following surgery. There is increasing evidence that many patients with COVID-19 are asymptomatic or have only mild symptoms, but are still able to transmit the virus to others [13]. Whether we are increasing their risk of asymptomatic infection following cataract surgery remains to be determined and requires further investigation.

Government reported data regarding the incidence of positive COVID-19 tests in the local government district of Ashford Hospital (Spelthorne) was 292 per 100,000 population at the start of the study (19 May 2020) and 457 per 100,000 population at the time of final data collection (17 September 2020). In comparison, the incidence at paper submission (14 Dec 2020) is now 2260 per 100,000 population [14].

We believe our study is robust due to our heterogenous group of patients, and thorough follow-up in which every patient or carer was contacted. Our cohort was comprised of people from BAME and non-BAME ethnicities, a broad age range and often with multiple comorbidities, including individuals potentially highly susceptible to COVID-19 infection. This is understandable as patients were not excluded from selection for cataract surgery on the basis of their health or comorbidities. One limitation is that we have not included data on patients who were offered cataract surgery but the patient declined to proceed, as this may reflect patients particularly at risk and hence unwilling to present to hospital except due to an emergency. Our hospital is also unusual in that we have no swab proven COVID-19 infected inpatients, potentially limiting applicability to hospitals with COVID-19 positive inpatients.

There was a restriction on anaesthetic service provision towards ophthalmology during the pandemic, due to redeployment of anaesthetists to other specialities, such as intensive care, and a higher demand in other ophthalmology sub-specialities necessitating emergency surgery under general anaesthesia, such as vitreoretinal and glaucoma surgery. There was a concerted effort by surgeons to perform cataract operations under local anaesthesia whenever possible. Hence the number of patients undergoing cataract surgery under general anaesthesia is particularly low in this study, with only three patients (0.5%) in total, which is not reflective of surgical numbers prior to COVID-19.

The study also sheds light on a concern regarding masks potentially increasing the rate of post-operative endophthalmitis. It has been suggested that exhaled air jets are directed towards the eye by masks [15]. Our cohort had no cases of endophthalmitis (and none up to the date of submission of this paper) despite all patients required to wear surgical masks following completion of the operation. As mentioned, all patients were issued with a new sterile face mask at the entrance of the hospital, which was removed just before draping and replaced following completion of the operation.

Our intra-operative complication rate was substantially lower than the national average. For example, posterior capsular rupture rate was 1.14% in the most recent NOD audit [1], compared to 0.6% in our study. The 2 month hiatus from surgery had the potential to impact surgical skills [16, 17]. However, we hypothesise that the reduced complications may be a result of the reduced time pressures the surgeons face, due to the staggered admission times and reduced numbers on the theatre lists, compared to pre-COVID-19 times. Alternatively, the limited general anaesthesia availability may have excluded particularly challenging cases. Conversely, surgeons may have operated on challenging cases under local anaesthesia which they may previously have operated on under general anaesthesia.

Similarly, our post-operative complication rate is also lower than expected [1]. It is likely that there has been under detection of post-operative complications, due to lack of routine face-to-face review and patients unwilling to present to hospital for further review. By collecting data at 30 day post-operatively, we will also miss later presenting complications, such as macular oedema.

One concern is a lack of clinic appointments, preventing patients from appropriately timed ophthalmic review in clinic, in response to post-operative symptoms. To counter this, we provided the patients with contact numbers within and outside of office hours, so they could speak directly with an ophthalmology-trained nurse or doctor relay their concerns. The vast majority of patients stated that they had no difficulty in arranging departmental review following the advent of their symptoms, adding support to routine telephone follow-up for uncomplicated cataract surgery. Longer term follow-up data is required to validate this model.

The recommended pre-operative isolation period changed during the study - from 14 days to 3 days, in keeping with NICE guidance [10]. Although not formally assessed, the majority of patients stated they complied with their quarantine period. The majority of patients in the study were instructed to isolate for 14 days, further studies are recommended to investigate the 3 day isolation period.

Lastly, with regards to subjective assessment, patients were in general very pleased with the service provided and happy they proceeded with cataract surgery. We appreciate this subjective assessment is prone to bias and hypothesise that patients may have underreported their concern for acquiring COVID-19 following the operation, in view of the fact they did not develop symptomatic infection. Patients concerned about acquiring COVID-19 infection would also have likely declined the offer of surgery. Hence we found a much lower proportion in comparison to pre-operative phone calls [18]. Similarly, we hypothesise that patients are more likely to retrospectively praise infection control measures, providing they were not knowingly infected.

We believe our study will be applicable for other ophthalmic procedures. We recommend further study of COVID-19 infection rates following ophthalmic surgery, including investigating asymptomatic infection and multi-centre studies encompassing hospitals with differing demographics and COVID-19 prevalence, including patients with COVID-19 positive inpatients.

In summary, we demonstrate the safety of resuming cataract surgery in a COVID-19 free hospital, which may be a necessary solution, particularly relevant as we head towards another ‘wave’ [19].

## Data Availability

The datasets used and/or analysed during the current study are available from the corresponding author on reasonable request.
